# Recovery of Fecal Microbiome and Bile Acids in Healthy Dogs after Tylosin Administration with and without Fecal Microbiota Transplantation

**DOI:** 10.3390/vetsci9070324

**Published:** 2022-06-27

**Authors:** Margaux Marclay, Elizabeth Dwyer, Jan S. Suchodolski, Jonathan A. Lidbury, Joerg M. Steiner, Frederic P. Gaschen

**Affiliations:** 1 Department of Veterinary Clinical Sciences, School of Veterinary Medicine, Louisiana State University, Baton Rouge, LA 70803, USA; mmarclay@medivetsa.ch (M.M.); elizabeth.dwyer@austinvets.com (E.D.); 2 Medi-Vet SA Vétérinaire, 1007 Lausanne, Switzerland; 3 Austin Veterinary Emergency and Specialty, Austin, TX 78730, USA; 4 Gastrointestinal Laboratory, Department of Small Animal Clinical Sciences, Texas A&M University, College Station, TX 77840, USA; jsuchodolski@cvm.tamu.edu (J.S.S.); jlidbury@cvm.tamu.edu (J.A.L.); jsteiner@cvm.tamu.edu (J.M.S.)

**Keywords:** fecal microbiome, fecal metabolome, dysbiosis, resilience, fecal bacteriotherapy

## Abstract

Antibiotics cause gut dysbiosis and bile acid dysmetabolism in dogs. The effect of fecal microbiota transplantation (FMT) on microbiome and metabolome recovery is unknown. This prospective, randomized, placebo-controlled study included sixteen healthy purpose-bred dogs. All dogs received tylosin 20 mg/kg PO once daily (days 1–7) and were randomly assigned to either receive one FMT via enema (day 8), daily oral FMT capsules (days 8–21), or daily placebo capsules (days 8–21). Fecal samples were frozen at regular intervals until day 42. Quantitative PCR for 8 bacterial taxa was performed to calculate the fecal dysbiosis index (FDI) and fecal concentrations of unconjugated bile acids (UBA) were measured using gas chromatography-mass spectrometry. Tylosin altered the abundance of most evaluated bacteria and induced a significant decrease in secondary bile acid concentrations at day 7 in all dogs. However, most parameters returned to their baseline by day 14 in all dogs. In conclusion, tylosin markedly impacted fecal microbiota and bile acid concentrations, although return to baseline values was quick after the antibiotic was discontinued. Overall, FMT did not accelerate recovery of measured parameters. Further studies are warranted to confirm the value of FMT in accelerating microbiota recovery in antibiotic-associated dysbiosis in dogs.

## 1. Introduction

The intestinal microbiota plays an important role in the development of the gut immune system, protects against pathogens, provides nutrients for the host, and aids in the development of the intestinal barrier [1]. In human medicine, alterations of the gut microbiome have been implicated in *Clostridioides difficile* infection, various other gastrointestinal diseases, and in other disorders, such as obesity, metabolic syndrome, and neurological and psychological diseases [2]. 

In dogs, manipulations of the microbiome are often attempted in order to treat acute and chronic intestinal diseases using antibiotics, prebiotics, probiotics, postbiotics and, more recently, fecal transplantation [3]. Various studies have evaluated the impact of antibiotics on the canine gut microbiome. Significant changes in fecal microbiota have been reported following the use of tylosin, metronidazole, and amoxicillin in healthy and sick dogs [4,5,6,7,8,9,10]. Tylosin is an antibiotic of the macrolide class, targeting aerobic Gram-positive bacteria, as well as some aerobic Gram-negative bacteria and select anaerobes [11]. In addition, tylosin is reported to have anti-inflammatory effects [12,13]. Recently, a prospective randomized study was performed on sixteen healthy adult dogs receiving either tylosin for 7 days or a placebo. The tylosin-exposed dogs exhibited a rapid decrease in fecal bacterial diversity and bile acids alterations. Some of these dogs experienced long-term changes and did not achieve a return to baseline microbiota composition after discontinuation of the antibiotic [7]. 

Large intestinal unconjugated bile acids (UBA) play an important role in host metabolism and interact with the gut immune system. Primary UBA are converted to secondary UBA by the large intestinal microbiota. *Clostridium hiranonis* was shown to have significant 7α-dehydroxylating activity and thereby contribute to conversion of primary into secondary UBA [1,14]. In people, altered intestinal UBA profiles with a decrease in secondary UBA have been linked to various gastrointestinal and hepatic diseases [15,16]. In dogs, secondary UBA concentrations were shown to be decreased with chronic enteropathy and exocrine pancreas insufficiency [17,18].

Fecal microbiota transplantation (FMT) and its effects on the intestinal microbiota have been the focus of much interest in recent years. FMT is now recognized as the treatment of choice for recurrent *Clostridioides difficile* infection in people and its beneficial effects in other gastrointestinal and extra-gastrointestinal diseases are the subject of ongoing studies [19,20]. While FMT is also used in dogs, there are few published studies evaluating its efficacy [21]. Puppies with parvovirus infection treated with FMT in addition to standard therapy had significantly shorter hospitalization and recovery times than controls [22]. Moreover, the fecal microbial and metabolic profiles of dogs with acute diarrhea receiving FMT improved while they did not in those treated with metronidazole [9]. 

The goal of our study was to evaluate the recovery of the fecal microbiome and fecal UBA profile of healthy dogs treated with tylosin after they were given FMT with oral capsules or enema in comparison to placebo. Our hypothesis was that FMT given via either route would accelerate the return to pre-tylosin values. The primary outcome was the fecal dysbiosis index and secondary outcomes included fecal abundances of bacterial taxa used to calculate the dysbiosis index and concentrations of fecal bile acids.

## 2. Materials and Methods

### 2.1. Animals

The study was approved by the Institutional Animal Care and Use Committee at Louisiana State University (Protocol 18-030) and performed in a facility accredited by the Association for Assessment and Accreditation of Laboratory Animal Care International. Sixteen healthy research dogs from the Louisiana State University School of Veterinary Medicine Division of Laboratory Animal Medicine (DLAM) were recruited. Eligible dogs were between 1 and 8 years of age. Exclusion criteria included: previous history of gastrointestinal disease and/or systemic antimicrobial exposure in the preceding 6 months. Dogs were initially screened by physical examination and laboratory diagnostics consisting of a CBC, serum biochemistry profile, fecal parasitology screening, including direct smear, flotation with centrifugation, and fecal enzyme-linked immunosorbent assay (ELISA) for Giardia detection. Fecal polymerase chain reaction (PCR) for enteropathogen detection (*Clostridioides difficile*, Canine parvovirus type 2, *Clostridium perfringens* enterotoxin gene, *Clostridium perfringens* NetF gene, *Campylobacter jejuni*, *Salmonella*) as well as immunofluorescence antibody (IFA) for Giardia and for Cryptosporidium detection were also performed at the GI Laboratory, College of Veterinary Medicine, Texas A&M University as part of the initial screening protocol. All dogs were given fenbendazole at 50 mg/kg PO q 24 h for 3 days prior to starting collection of feces for the study period.

A healthy donor was selected from the sixteen healthy research dogs. All results from the previously mentioned laboratory tests were within the respective reference intervals prior to enrolling this dog as a donor. The donor was screened to rule out dysbiosis using the qPCR-based fecal dysbiosis index as previously described [23]. The donor was selected based on a fecal dysbiosis index (FDI) <0 and *C. hiranonis* qPCR within the reference interval. The donor dog was later randomized to the placebo group.

### 2.2. Fecal Microbiota Transplantation Preparation

The donor feces were collected from the cage floor within 12 h of defecation for several days before the beginning of the study and prepared for FMT within 4 h of collection. Feces were processed as follows: 4 volumes of 0.9% saline were added to 1 volume of feces, resulting in a 20% fecal solution, which was mixed and filtered in a two-compartment plastic bag with a filter layer of finely perforated polyethylene, to remove large particles (Whirl-Pak Homogenizer Blender Filter Bag, Sigma Aldrich Corp., St. Louis, MO, USA). Then, 11 mL of 100% glycerol was added to 100 mL of the fecal solution before it was frozen in aliquots at −80 °C and stored for up to 2 months. For FMT administered via enema, the frozen fecal solution was thawed in a 37 °C water bath and instilled within 4 h into the proximal descending colon at a dose of 10 mL/kg. For oral FMT, the fecal solution described above was centrifuged, the supernatant discarded, and the final sediment (600 μL) pipetted into #00 gelatin capsules that were over encapsulated with #000 gelatin capsules. For the placebo group, 1 volume glycerol was added to 9 volumes of 0.9% saline and 600 μL of the solution was pipetted into the same gelatin capsules that were also over encapsulated. The fecal and placebo capsules were then frozen and stored at −80 °C for up to 2 months until use. In the absence of recommendations for oral FMT in dogs, we adapted a dose used in a recent study evaluating oral FMT in human patients [24]. Accordingly, each dog was given oral capsules daily for 2 weeks, corresponding to a total dose of approximately 1 g feces per kg BW administered over the 2-week period. 

### 2.3. Study Design 

**Figure 1:**
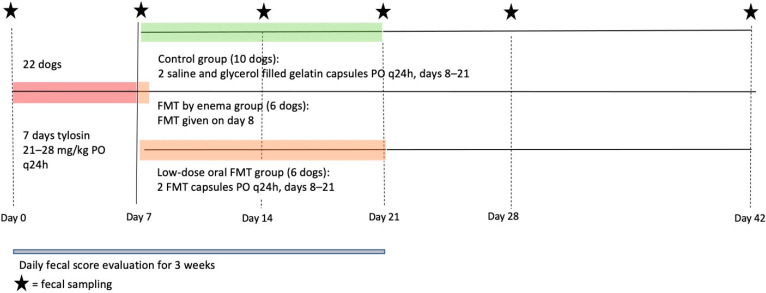
Schematic illustrating study intervention, duration, observations, and sampling. Legend: FMT; fecal microbiota transplantation.

This was a prospective, randomized, placebo-controlled study and its design is illustrated in Figure 1. Dogs were paired based on the existing housing plan (indoor runs), and each dog pair was given a number and randomly assigned to one of three separates treatment groups: placebo (group 1, 10 dogs; 4 concurrent with the FMT dogs and 6 FMT dogs that were re-enrolled as placebo dogs one year later), enema FMT (group 2, 6 dogs), and oral FMT (group 3, 6 dogs). All sixteen dogs received a 7-day course of oral tylosin at approximately 20 mg/kg PO q24h (median dose 23.1 mg/kg; minimum to maximum dose 21.1–27.8 mg/kg). 

On day 8 (D8), group 2 received a single FMT by enema at a dose of 10 mL/kg. The dogs were sedated with butorphanol (0.3 mg/kg IV) and dexmedetomidine (0.004 mg/kg IV) prior to the procedure and maintained in lateral recumbency for 30 min following the enema. Additionally, starting on the same day, group 3 received the fecal gelatin capsules PO once daily for 2 weeks (median dose 2.6 capsules per day; range: 2–4). Finally, group 1 received 2 placebo gelatin capsules PO once daily for 2 weeks. 

Feces from each dog were collected at six time points: at the beginning of the study prior to antibiotic treatment (D0), on the last day of antibiotic treatment (D7), then weekly for 3 weeks (D14, D21, and D28), and finally 6 weeks after starting antibiotics (D42). Feces were placed in plastic vials and immediately frozen and stored at −80 °C. They were then shipped as a batch on dry ice and analyzed within 3 months of collection at the GI laboratory, College of Veterinary Medicine, Texas A&M University. The consistency of each dog’s feces was evaluated daily for 3 weeks and each time the feces were collected using a 5-point pictorial fecal scoring system (Fecal Scoring System for Dogs, Royal Canin, Aimargues, France). 

### 2.4. DNA Extraction and Quantitative PCR

All samples underwent 1 freeze–thaw cycle before DNA extraction. Fecal DNA was extracted from a 100 mg aliquot of feces using the DNeasy PowerSoil Kit (QIAGEN Inc., Germantown, MD, USA) according to the manufacturer’s instructions. Quantitative PCR was then used to quantify the abundance of selected bacterial groups (Universal, *Blautia* spp., *C. hiranonis*, *E. coli*, *Faecalibacterium* spp., *Fusobacterium* spp., *Streptococcus* spp., *Turicibacter* spp.) on all fecal samples (*n* = 144) as previously reported [23]. Results were expressed as the log amount of DNA (fg) for each particular bacterial group per 10 ng of total isolated DNA. A mathematical algorithm was employed to convert these abundances into a single descriptive numeric value, the FDI [23].

### 2.5. Fecal Bile Acid Concentrations

The concentrations of fecal cholic acid (CA), chenodeoxycholic acid (CDCA), lithocholic acid (LCA), deoxycholic acid (DCA), and ursodeoxycholic acid (UDCA) were measured in lyophilized feces using a dilution gas chromatography-mass spectrometry (GC-MS) method as previously reported in all samples collected [18]. Concentrations of primary (CA and CDCA) and secondary (DCA, LCA, and UDCA) UBA were combined and expressed as primary UBA and secondary UBA. Results were reported as micrograms per milligram of lyophilized feces and percentage of total UBA pool. 

### 2.6. Statistical Analysis

Descriptive statistics were calculated for each variable. Data and residuals were assessed for normality using the Shapiro–Wilk test. Data that did not meet normality were log transformed, and log data and residuals were retested using the Shapiro–Wilk test. Data was expressed as mean and standard deviation or median and range (minimum-maximum) if it was not normally distributed prior to log transformation. 

Fecal scores were evaluated within groups at the different time points with a Friedman 2-way ANOVA for non-parametric data. Comparison of dysbiosis index, qPCR for selected bacterial taxa, total, primary and secondary BAs within groups was performed using 2-way repeated-measures ANOVA based on general linear model with Geisser-Greenhouse correction when sphericity was not met (Epsilon < 0.75) and subsequent post hoc comparison (Dunnett). Fixed factors included treatment, time and treatment-by-time interaction. A *p*-value < 0.05 was considered statistically significant. Statistical analyses were performed using a commercially available software package (Prism 9 for macOS version 9.1.1, Graph Pad Software, San Diego, CA, USA).

## 3. Results

### 3.1. Fecal Scores and Dog Observation

The fecal scores for all 3 groups remained between 3.5 (“moist stool starting to lose its shape and cracks”) and 4 (“stool with clearly defined shape and visible cracks, leaves little residue on the ground when picked up”) during the initial daily 21-day observation period (D1-21), as well as on D28 and D42. There were no significant differences between time points. All dogs receiving FMT via enema experienced one episode of diarrhea within 24 h of the procedure, which resolved without any intervention. One dog receiving FMT via oral capsules vomited once, which was later avoided by administering a small amount of food with the capsules. 

Over the course of the study, several dogs across all 3 groups exhibited coprophagic behavior. However, since the dogs were kept in pairs that were assigned to the same group and their pens were cleaned daily, they could only eat their own feces from the same day, or those of their kennelmate.

### 3.2. Abundance of Bacterial Taxa and Fecal Dysbiosis Index

**Figure 2:**
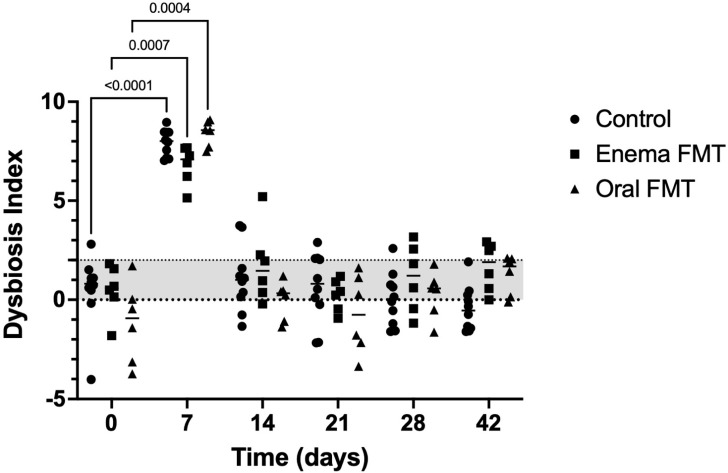
Dot plot graph representing the variation of the fecal dysbiosis index in control dogs, dogs receiving FMT by enema, and dogs receiving FMT orally over the 6-week duration of the study. Legend: Fecal dysbiosis index (FDI) < 0 is considered normal while a value between 0 and 2 is considered to be equivocal, and FDI > 2 is abnormal. Dots represent individual values for each dog, bars denote median for the group. Statistically significant pairwise comparisons are shown.

The median initial FDIs of the control and enema FMT groups were both in the equivocal range while that of the oral FMT dogs was in the normal range. Overall, FDI increased above 2 during treatment with tylosin in all groups (*p* < 0.001) and normalized 7 days after the antibiotic was discontinued in all dogs. Several dogs from the control and enema FMT groups had an abnormal value (>2) at different time points between D14 and D42 (Figure 2).

**Figure 3:**
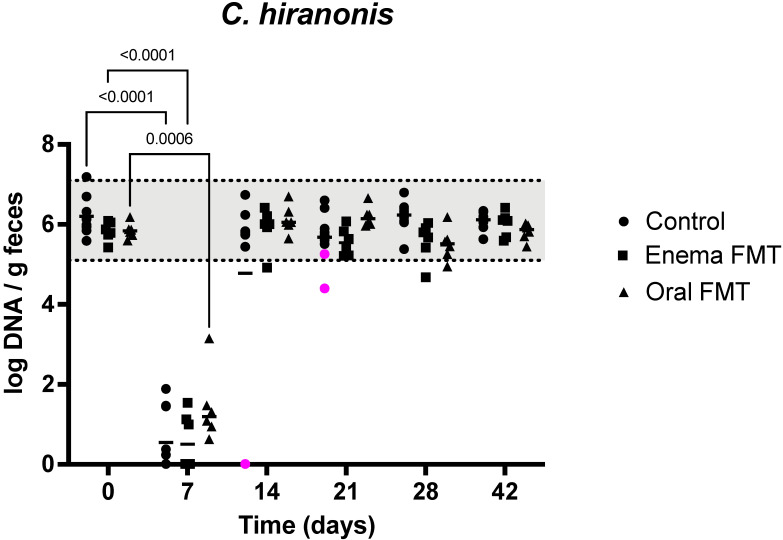
Dot plot graph representing the variation of fecal *C. hiranonis* abundance in control dogs, dogs receiving FMT by enema, and dogs receiving FMT orally over the 6-week duration of the study. Legend: The shaded area highlights the reference interval for the abundance of *C. hiranonis* in feces from dogs Dots represent individual values for each dog, bars denote median for the group and time point. Statistically significant pairwise comparisons are shown. The magenta dot identifies 2 dogs with identicallyvery low fecal abundances of *C. hiranonis* at day 14 that also had low secondary fecal bile acid concentrations at that same time (see Figure 2). The abundance remained below the reference interval in one dog at day 21.

**Figure 4:**
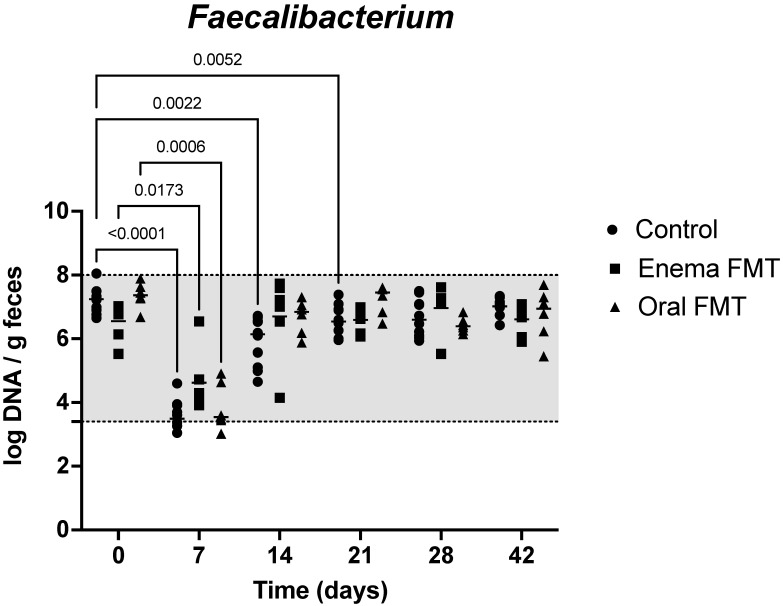
Dot plot graph representing the variation of fecal *Faecalibacterium* abundance in control dogs, dogs receiving FMT by enema, and dogs receiving FMT orally over the 6-week duration of the study. Legend: The shaded area highlights the reference interval of the abundance for *Faecalibacterium* in dog feces. Dots represent individual values for each dog, bars denote median for the group and time point. Statistically significant pairwise comparisons with baseline in each group are shown.

Results of quantitative PCR evaluation of 7 bacterial taxa in the feces for the first 14 days of the study are shown in Table 1. *Blautia* abundance was mildly decreased at D7, but recovered to initial values by D14 in controls, while it was unchanged at D7 in dogs receiving FMT. However, *Blautia* abundance was decreased at D14, 7 days after discontinuation of tylosin and FMT administration in enema FMT dogs (*p* = 0.0013). It was also decreased at D21 after 14 days of treatment with oral capsules (6.90; 5.88–7.59 [median; range]) when compared to initial abundances (10.2; 9.6–10.6) in oral FMT dogs (*p* = 0.009). It remained unchanged from the baseline at all other times (Data not shown). *C. hiranonis* abundance severely decreased at D7 but recovered at day 14 in all but two dogs. These two control dogs had severely decreased abundances of 0.01 log DNA/g feces at D7 and D14 that remained persistently low at D21 and recovered to initial values at D28 (Figure 3). *E. coli* abundance increased at D7 but normalized at D14 in control dogs and oral FMT dogs. It was not significantly increased at D7 in enema FMT dogs (*p* = 0.10). *Faecalibacterium* abundance was significantly decreased while remaining in the lab’s reference interval at D7 in all dogs and recovered by D14 in dogs receiving FMT by enema or oral capsules. It was still decreased at D14 and D21 in control dogs and only recovered to initial values at D28 (Figure 4). *Fusobacterium* and *Turicibacter* abundances decreased at D7 and recovered at D14 in all dogs. Finally, *Streptococcus* abundances remained unchanged in controls and enema FMT dogs while they increased at D7 but recovered to initial values at D14 in oral FMT dogs.

### 3.3. Fecal Bile Acid Concentrations

**Figure 5:**
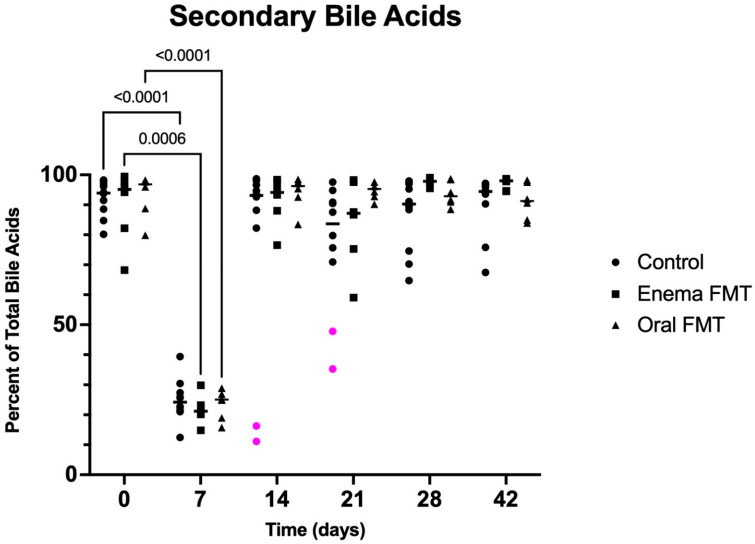
Dot plot graph representing the percentage of secondary fecal UBA in control dogs, dogs receiving FMT by enema, and dogs receiving FMT orally over the 6-week duration of the study. Legend: Dots represent individual values for each dog, bars denote medians for the group and time point. Statistically significant pairwise comparisons are shown. The magenta dots identify 2 dogs with low percentual secondary fecal bile acid concentrations at day 14 that also had very low fecal abundances of *C. hiranonis* at that same time (see Figure 3). Percentual secondary bile acid concentrations were also low at day 21 in these 2 dogs.

Data on fecal bile acid concentrations for the first 14 days of the study are summarized in Table 2. Total primary and percentual fecal UBA concentrations were markedly increased on D7 in all groups when compared to baseline. They normalized in all dogs one week later. Conversely, total and percentual secondary fecal UBA concentrations were severely decreased on the last day of tylosin treatment (D7) in all groups when compared to baseline. However, they returned to baseline at D14 and were not different from baseline in any of the following sampling points in any group (Figure 5). The two control dogs with continuously decreased C. hiranonis concentrations at D14 and D21 also maintained a high percentage of primary bile acids (D14: 83.7 and 88.9, resp; D21: 64.7 and 52.8, resp.) and a low percentage of secondary bile acids (D14: 16.3 and 11.1, resp.; D21: 35.3 and 47.2, resp.) at these time points.

## 4. Discussion

The main results of our study can be summarized as follows: first, the immediate impact of tylosin on the fecal microbiome and bile acid profile of healthy dogs was confirmed, although these effects were not as long-lasting as had previously been documented [4,7]. Second, only marginal positive differences in microbiome recovery could be detected in the feces of dogs receiving FMT.

Two previous studies reported on the effects of tylosin on the fecal microbiome and metabolome of dogs. Suchodolski et al. (2009) administered tylosin at the same dose as we did (20–25 mg/kg PO q24h) to purpose-bred dogs with a pre-existing jejunal fistula [4]. They sampled brushings from the jejunal mucosa before, during and 14 days after antibiotic treatment to assess variations in the mucosa-adherent microbiota using pyrosequencing. They noticed decreases in numerous bacterial taxa and a relative increase in inherently tylosin-resistant *E. coli* (which was also increased in the present study) and *C. perfringens* (which we did not evaluate) on the last day of treatment that in some dogs persisted for another 2 weeks. That study focused on mucosa-associated small intestinal microbiota while our study investigated the luminal large intestinal microbiota. The impact of the same dose of tylosin might have been different in these separate microbial communities, ref. [1] moreover the techniques used varied widely. Therefore, the results of the 2 studies are not easily comparable. Manchester et al. (2019) administered tylosin at a daily dose twice as high as the dose we used to healthy pet dogs recruited from a teaching hospital community [7]. Like in our study, qPCR analysis showed a major impact of the antibiotic at the end of treatment with decreases in abundances of *Blautia*, *C. hiranonis*, *Faecalibacterium*, *Fusobacterium*, and *Turicibacter* with a recovery of most of these taxa 2 weeks after discontinuation of the antibiotic. However, *Fusobacterium* only returned to normal abundances 8 weeks after tylosin was stopped. In addition, no effect of tylosin on *E. coli* abundance was documented, unlike in the present study where it was increased at the end of tylosin treatment in 2 of the 3 groups but back to initial values 1 week later. Additionally, the FDI in their dogs remained increased compared to baseline 2 weeks after tylosin discontinuation and was back to initial values at their next measurement 6 weeks later. However, the authors did not provide any data on the evolution of the fecal microbiota between 2 and 8 weeks after discontinuation of tylosin, and this makes a close comparison of outcomes between the 2 studies difficult. 

In another recently published study, Pilla et al. investigated the effects of a 2-week metronidazole treatment on the fecal microbiota of healthy pet dogs [8]. The FDI significantly increased during the 2 weeks of antibiotic treatment, but was not significantly higher than initial values at 2 and 4 weeks after discontinuation of the antibiotic, even though the median values remained in the equivocal range and some dogs had persistent dysbiosis as indicated by an FDI > 2. As was the case with tylosin in the present study, abundances of *C. hiranonis*, *Faecalibacterium*, *Fusobacterium*, and *Turicibacter* were all decreased during metronidazole treatment while those of *E. coli* and *Streptococcus* were increased. However, abundances of these 7 bacterial taxa were not different from the initial values 2 weeks after discontinuation of antibiotics. Unlike our study, the study by Pilla et al. did not evaluate the fecal microbiota 1 week following treatment cessation. 

Both the Manchester et al. and the Pilla et al. papers included more in-depth evaluation of the fecal microbiome through sequencing of the 16S rRNA genes [7,8]. Alpha-diversity with species richness and diversity were both decreased during antibiotic administration and back to baseline 2–8 weeks later. However, beta-diversity (diversity between samples) remained significantly different from baseline up to 8 weeks after antibiotics were discontinued. In addition, a shift in microbial communities of the fecal samples was reported for all sampling times persisting for up to 4–8 weeks after antibiotics were discontinued. Sequencing was not performed in the present study and might have demonstrated additional differences between controls and FMT dogs.

A 3rd study evaluated the effects of amoxicillin with or without clavulanic acid on dogs prescribed a 5-to-7-day course of these antibiotics for various diseases in 2 companion animal clinics [10]. Fecal samples were collected during and 1 week after discontinuation of treatment. Alpha-diversity of the fecal microbiota was decreased during treatment followed by a return to pre-treatment conditions 1 week after completion of the treatment. In addition, a rise in ampicillin-resistant *E. coli* and *Enterococci* occurred during treatment [10]. The latter finding was confirmed in a study of dogs with acute diarrhea given amoxicillin and clavulanic acid for 7 days [25]. 

In the present study, total and percentual fecal concentrations of primary UBA were increased while those of secondary UBA were decreased at the end of tylosin treatment and recovered 1 week after tylosin discontinuation. This is similar to what happened following metronidazole administration [8]. However, the paper by Manchester et al. showed an increase in fecal concentrations of primary UBA that persisted for 8 weeks after antibiotic discontinuation [7]. Total concentrations of fecal UBA did not vary in any of these studies. A strong correlation between *C. hiranonis* and secondary fecal UBA has previously been described in dogs, as *C. hiranonis* expresses a high 7α-dehydroxylation activity, which is related to the conversion of primary to secondary UBA [8,26,27]. The latter have the ability to modulate the composition of the gut microbiota both directly and indirectly via activation of the innate immune system, and to affect the host metabolism through signaling via bile acid receptors [16,28]. Therefore, a decrease in fecal concentrations of secondary UBA as occurs in healthy dogs receiving antibiotics has the potential to further contribute to gut dysbiosis and compromise homeostasis of the host metabolism. 

The stronger resilience of the fecal microbiota to tylosin in our study could be due to the different tylosin dosing regimen used. Pharmacokinetic studies for oral administration are not available, however a dose of 10 mg/kg IV q12 to 24 h was reported to be efficacious against sensitive *Pasteurella* and hemolytic *Staphylococcus* spp. in dogs [29]. In addition, doses as low as 5 mg/kg PO were successful in the treatment of tylosin-responsive chronic diarrhea, presumably through modulation of the gut microbiome [30]. Tylosin doses for oral administration listed in pharmacotherapy textbooks vary from 5 to 80 mg/kg with a dosing interval from one to three times daily [11]. In addition, the fact that our dogs were part of a teaching colony and not pet dogs as in recent studies investigating the effects of antibiotics on the fecal microbiota may also have influenced the resilience of their gut microbiota [7,8,10]. Environmental factors such as diet may play a role as was demonstrated in a recent study in which host diet had a major impact on the response of specific bacterial taxa to antibiotic administration in mice [31]. Dogs kept in a colony setting experience a more controlled lifestyle including a consistent level of activity and diet than pet dogs do, and it is conceivable that this stable environment may positively influence the resilience of their gut microbiome to short-term perturbations [32]. However, this hypothesis is not supported by the prolonged (2 weeks or more) effect of tylosin on several bacterial taxa of the mucosa-adherent microbiota in part of the laboratory beagles with jejunal fistula [4]. Finally, some dogs included in our study had coprophagic behavior, which might represent another confounding factor. Studies in small rodents have shown that preventing coprophagy can significantly alter their small and large intestinal microbiome and metabolome with possible impacts on their health [33,34]. Our dogs were held in pairs and both dogs were assigned to the same treatment group to minimize the potential interference of coprophagy on the study. However, it is impossible to rule out that coprophagy somehow influenced the resilience of their fecal microbiome.

We found FMT to be a well-tolerated procedure. The dose of fecal solution per kg recipient BW for FMT via enema was adapted from a study performed in human patients and equivalent to approx. 2 g of donor feces per kg BW [24]. Since then, others have used higher doses of 2.5–5 g of feces per kg with success in clinical cases [9]. However, an ideal dose of FMT given via enema to dogs has not been determined to date [21]. Additionally, we adapted the oral dose of FMT to 1 g of donor feces per kg BW distributed over a 2 week-period. In our study, the donor feces were left at room temperature for up to 12 h. While there is no data for canine feces, storage at ambient temperature of human and feline feces did not affect numbers of operational taxonomic units, diversity or richness of the fecal microbiome [35,36]. 

The changes in the abundances of most tested bacterial taxa were short lived in all dogs, however, abundance of *Faecalibacterium* in control dogs was significantly decreased from baseline for 2 weeks following discontinuation of tylosin but remained within the lab’s reference interval. This persisting change was not observed in dogs receiving FMT via enema or orally which only showed a decreased abundance of *Faecalibacterium* at the end of the tylosin treatment. *Faecalibacterium* produces anti-inflammatory peptides [37]. and short-chain fatty acids (SCFA) from dietary carbohydrates, and SCFA act as nutrients for enterocytes, and have many additional properties to maintain gut health [3]. However, taken together, these findings do not demonstrate a clear positive effect of FMT in dogs suffering from tylosin induced gut dysbiosis. We cannot rule out that fluctuations in *Faecalibacterium* abundances might have occurred randomly as was apparently the case for *Blautia* at select time points. Importantly, the model used in the present study was not optimal to evaluate the effects of FMT since most of the measured microbiome and metabolome parameters normalized rapidly after discontinuation of tylosin. 

Our study did not include a full analysis of the fecal microbiome and metabolome. Therefore, we did not obtain any data on alpha- and beta-diversity of the microbiome nor on bacterial phyla and taxa beyond those we reported on. However, previous studies have shown good correlation between FDI and alpha and beta diversity [8,27]. Additionally, our metabolomic analysis was limited to fecal UBAs. Additional data might have revealed a more profound and long-lasting effect of antibiotic treatment and shown an improvement of these parameters following FMT. 

In summary, the present study replicated most of the changes in fecal microbiota and bile acids that had been documented in recent studies using tylosin, metronidazole, or amoxicillin and clavulanic acid in healthy dogs. However, most of these parameters returned to baseline values 1 week after discontinuation of antibiotics, and this suggests that the duration of the impact of oral antibiotics may vary based on the dosing regimen and the dogs’ environment and lifestyle. In addition, FMT administered via enema or orally was well tolerated. However, given the return to baseline of the FDI and UBA after the antibiotics were discontinued, it did not accelerate recovery of these parameters. These preliminary results should be the basis of further studies evaluating the effect of FMT following spontaneous or iatrogenic perturbations of the gut microbiome in dogs. 

## Figures and Tables

**Table 1:** Results of qPCR analysis of 7 bacterial taxa in the feces at baseline (D0), end of tylosin treatment (D7) and 1 week after tylosin discontinuation (D14).

	Universal	*Blautia*	*C. hiranonis*	*Faecalibacterium*	*Fusobacterium*	*E. coli*	*Streptococcus*	*Turicibacter*
Reference interval ^#^	10.6–11.4	9.5–11.0	5.1–7.1	3.4–8.0	7.0–10.3	0.9–8.0	1.9–8.0	4.6–8.1
Control (D0)	11.2 (11.0–11.4)	10.4 (10.0–10.9)	6.1 (5.6–7.2)	7.2 (6.7–8.1)	9.1 (8.5–10.1)	7.3 (5.1–7.6)	8.2 (4.8–8.5)	7.4 (6.1–8.2)
Control (D7)	11.1 (10.5–11.4)	9.8 * (8.7–10.2)	0.13 * (0.0–1.9)	3.5 * (3.1–4.6)	7.6 * (7.0–8.0)	8.5 * (7.9–8.9)	7.6 (6.5–8.4)	5.2 * (4.8–5.6)
Control (D14)	11.0 (10.2–11.4)	10.2 (9.3–10.6)	5.8 (0.0–6.7)	6.1* (4.7–6.7)	9.4 (8.7–9.7)	7.1 (6.2–7.7)	7.3 (5.3–8.3)	7.2 (5.5–8.1)
Enema FMT (D0)	10.5 (10.4–11.0)	10.0 (9.8–10.1)	5.8 (5.4–6.0)	6.8 (5.5–7.0)	8.7 (8.4–9.3)	6.6 (5.6–7.6)	8.0 (5.7–8.8)	7.2 (6.7–8.0)
Enema FMT (D7)	10.7 (10.7–10.8)	10.0 (9.9–10.5)	0.5 * (0–1.5)	4.2 * (3.9–6.5)	7.7 * (7.4–8.2)	8.0 (7.8–8.8)	7.9 (7.7–8.6)	5.2 * (4.8–5.5)
Enema FMT (D14)	11.1 * (11.0–11.3)	7.5 * (6.4–8.2)	6.0 (4.9–6.4)	7.1 (4.2–7.7)	8.8 (7.8–9.6)	7.5 (6.4–8.2)	8.5 (7.7–8.9)	7.6 (6.0–8.6)
Oral FMT (D0)	11.2 (11.1–11.2)	10.2 (9.6–10.6)	5.8 (5.6–6.2)	7.4 (6.7–7.9)	9.3 (9.0–9.6)	5.9 (5.1–6.7)	6.7 (4.8–7.8)	7.5 (6.2–8.1)
Oral FMT (D7)	11.1 (11.0–11.3)	9.5 (6.9–10.3)	1.2 * (0.6–3.1)	3.5 * (3.0–4.9)	7.1 * (6.8–7.5)	8.2 * (8.0–8.5)	8.7 * (8.4–8.9)	5. 2 * (5.1–5.5)
Oral FMT (D14)	11.1 (10.9–11.3)	10.4 (9.9–11.1)	6.1 (5.6–6.7)	6.8 (5.9–7.3)	9.6 (8.8–10.0)	6.8 (5.5–7.7)	7.6 (6.3–8.1)	7.9 (5.9–8.2)

All data expressed as median (minimum—maximum) log DNA/gram of feces. # Texas A&M College of Veterinary Medicine GI Laboratory Reference Intervals for fecal qPCR analysis of 7 bacterial taxa. D0 = baseline, D7 = last day of tylosin treatment, D14 = 7 days after discontinuation of tylosin. * Statistically different from D0 in same group.

**Table 2:** Fecal concentrations of total UBA and total and relative fecal concentrations of primary and secondary UBA at baseline (D0), end of tylosin treatment (D7) and 1 week after tylosin discontinuation (D14).

	Total UBA Concentration (μg/mg)	Primary UBA Concentration (μg/mg)	Primary UBA Percentage of Total UBA	Secondary UBA Concentration (μg/mg)	Secondary UBA Percentage of Total UBA
Control (D0)	2.65 (1.88–4.27)	0.16 (0.05–0.72)	6.1 (1.7–19.8)	2.51 (0.52–4.00)	93.4 (80–2-98.3)
Control (D7)	1.79 (1.01–5.03)	1.40 (0.61–4.41) *	75.8 (60.7–87.5) *	0.42 (0.33–0.63) *	24.2 (12.5–39.3) *
Control (D14)	3.38 (1.34–7.31)	0.24 (0.07–3.45)	6.9 (1.3–88.9)	2.41 (0.43–7.07)	93.1 (11.1–98.7)
Enema FMT (D0)	2.46 (1.18–5.75)	0.15 (0.03–0.49)	4.9 (0.5–31.8)	2.20 (0.80–5.72)	95.1 (68.2–99.4)
Enema FMT (D7)	2.02 (1.37–4.01)	1.56 (1.09–3.37) *	78.8 (70.1–85.2) *	0.46 (0.28–0.81) *	21.2 (14.8–29.9) *
Enema FMT (D14)	4.00 (1.26–4.39)	0.16 (0.03–0.88)	5.9 (1.6–23.4)	3.34 (1.23–4.32)	94.1 (76.6–98.4)
Oral FMT (D0)	3.15 (1.09–4.01)	0.11 (0.04–0.79)	3.1 (1.7–20.1)	3.02 (0.97–3.92)	96.9 (79.9–98.9)
Oral FMT (D7)	2.14 (1.44–3.61)	1.69 (1.08–2.70) *	74.9 (71.1–84.3) *	0.53 (0.35–0.91)*	25.1 (15.7–28.9) *
Oral FMT (D14)	3.33 (2.14–4.81)	0.16 (0.06–0.35)	3.7 (1.5–16.6)	3.19 (1.79–4.70)	96.3 (83.4–98.5)

All data expressed as median (minimum–maximum). D0 = baseline, D7 = last day of tylosin treatment, D14 = 7 days after discontinuation of tylosin. * Statistically different from D0 in same group.

## Data Availability

The data presented in this study are available on request from the corresponding author. The data are not publicly available due to institutional policies.

## References

[1] Pilla R., Suchodolski J.S. (2019). The Role of the Canine Gut Microbiome and Metabolome in Health and Gastrointestinal Disease. Front. Vet. Sci..

[2] Durack J., Lynch S.V. (2019). The gut microbiome: Relationships with disease and opportunities for therapy. J. Exp. Med..

[3] Ziese A.L., Suchodolski J.S. (2021). Impact of Changes in Gastrointestinal Microbiota in Canine and Feline Digestive Diseases. Vet. Clin. N. Am. Small Anim. Pract..

[4] Suchodolski J.S., Dowd S.E., Westermarck E., Steiner J.M., Wolcott R.D., Spillmann T., Harmoinen J.A. (2009). The effect of the macrolide antibiotic tylosin on microbial diversity in the canine small intestine as demonstrated by massive parallel 16S rRNA gene sequencing. BMC Microbiol..

[5] Gronvold A.M., L’Abee-Lund T.M., Sorum H., Skancke E., Yannarell A.C., Mackie R.I. (2010). Changes in fecal microbiota of healthy dogs administered amoxicillin. Fems. Microbiol. Ecol..

[6] Igarashi H., Maeda S., Ohno K., Horigome A., Odamaki T., Tsujimoto H. (2014). Effect of oral administration of metronidazole or prednisolone on fecal microbiota in dogs. PLoS ONE.

[7] Manchester A.C., Webb C.B., Blake A.B., Sarwar F., Lidbury J.A., Steiner J.M., Suchodolski J.S. (2019). Long-term impact of tylosin on fecal microbiota and fecal bile acids of healthy dogs. J. Vet. Intern. Med..

[8] Pilla R., Gaschen F.P., Barr J.W., Olson E., Honneffer J., Guard B.C., Blake A.B., Villanueva D., Khattab M.R., AlShawaqfeh M.K. (2020). Effects of metronidazole on the fecal microbiome and metabolome in healthy dogs. J. Vet. Intern. Med..

[9] Chaitman J., Ziese A.L., Pilla R., Minamoto Y., Blake A.B., Guard B.C., Isaiah A., Lidbury J.A., Steiner J.M., Unterer S. (2020). Fecal Microbial and Metabolic Profiles in Dogs With Acute Diarrhea Receiving Either Fecal Microbiota Transplantation or Oral Metronidazole. Front. Vet. Sci..

[10] Espinosa-Gongora C., Jessen L.R., Kieler I.N., Damborg P., Bjornvad C.R., Gudeta D.D., Dos Santos T.P., Sablier-Gallis F., Sayah-Jeanne S., Corbel T. (2020). Impact of oral amoxicillin and amoxicillin/clavulanic acid treatment on bacterial diversity and beta-lactam resistance in the canine faecal microbiota. J Antimicrob Chemother.

[11] Budde J.A. (2022). Tylosin drug monograph. Plumb’s Veterinary Drugs Online.

[12] Cao X.Y., Dong M., Shen J.Z., Wu B.B., Wu C.M., Du X.D., Wang Z., Qi Y.T., Li B.Y. (2006). Tilmicosin and tylosin have anti-inflammatory properties via modulation of COX-2 and iNOS gene expression and production of cytokines in LPS-induced macrophages and monocytes. Int. J. Antimicrob. Agents.

[13] Menozzi A., Pozzoli C., Poli E., Lazzaretti M., Cantoni A., Grandi D., Giovannini E., Coruzzi G. (2005). Effect of the macrolide antibacterial drug, tylosin, on TNBS-induced colitis in the rat. Pharmacology.

[14] Kitahara M., Takamine F., Imamura T., Benno Y. (2001). *Clostridium hiranonis* sp. nov., a human intestinal bacterium with bile acid 7alpha-dehydroxylating activity. Int. J. Syst. Evol. Microbiol..

[15] Ramirez-Perez O., Cruz-Ramon V., Chinchilla-Lopez P., Mendez-Sanchez N. (2017). The Role of the Gut Microbiota in Bile Acid Metabolism. Ann. Hepatol..

[16] Wahlstrom A., Sayin S.I., Marschall H.U., Backhed F. (2016). Intestinal Crosstalk between Bile Acids and Microbiota and Its Impact on Host Metabolism. Cell Metab..

[17] Blake A.B., Guard B.C., Honneffer J.B., Lidbury J.A., Steiner J.M., Suchodolski J.S. (2019). Altered microbiota, fecal lactate, and fecal bile acids in dogs with gastrointestinal disease. PLoS ONE.

[18] Guard B.C., Honneffer J.B., Jergens A.E., Jonika M.M., Toresson L., Lawrence Y.A., Webb C.B., Hill S., Lidbury J.A., Steiner J.M. (2019). Longitudinal assessment of microbial dysbiosis, fecal unconjugated bile acid concentrations, and disease activity in dogs with steroid-responsive chronic inflammatory enteropathy. J. Vet. Intern. Med..

[19] Borody T.J., Eslick G.D., Clancy R.L. (2019). Fecal microbiota transplantation as a new therapy: From Clostridioides difficile infection to inflammatory bowel disease, irritable bowel syndrome, and colon cancer. Curr. Opin. Pharmacol..

[20] Zhou Y., Xu H., Huang H., Li Y., Chen H., He J., Du Y., Chen Y., Zhou Y., Nie Y. (2019). Are There Potential Applications of Fecal Microbiota Transplantation beyond Intestinal Disorders?. Biomed Res. Int..

[21] Chaitman J., Gaschen F. (2021). Fecal Microbiota Transplantation in Dogs. Vet. Clin. N. Am. Small Anim. Pract..

[22] Pereira G.Q., Gomes L.A., Santos I.S., Alfieri A.F., Weese J.S., Costa M.C. (2018). Fecal microbiota transplantation in puppies with canine parvovirus infection. J. Vet. Intern. Med..

[23] AlShawaqfeh M.K., Wajid B., Minamoto Y., Markel M., Lidbury J.A., Steiner J.M., Serpedin E., Suchodolski J.S. (2017). A dysbiosis index to assess microbial changes in fecal samples of dogs with chronic inflammatory enteropathy. Fems. Microbiol. Ecol..

[24] Kao D., Roach B., Silva M., Beck P., Rioux K., Kaplan G.G., Chang H.J., Coward S., Goodman K.J., Xu H. (2017). Effect of Oral Capsule- vs Colonoscopy-Delivered Fecal Microbiota Transplantation on Recurrent Clostridium difficile Infection: A Randomized Clinical Trial. JAMA.

[25] Werner M., Suchodolski J.S., Straubinger R.K., Wolf G., Steiner J.M., Lidbury J.A., Neuerer F., Hartmann K., Unterer S. (2020). Effect of amoxicillin-clavulanic acid on clinical scores, intestinal microbiome, and amoxicillin-resistant Escherichia coli in dogs with uncomplicated acute diarrhea. J. Vet. Intern. Med..

[26] Wang S., Martins R., Sullivan M.C., Friedman E.S., Misic A.M., El-Fahmawi A., De Martinis E.C.P., O’Brien K., Chen Y., Bradley C. (2019). Diet-induced remission in chronic enteropathy is associated with altered microbial community structure and synthesis of secondary bile acids. Microbiome.

[27] Li Q., Larouche-Lebel E., Loughran K.A., Huh T.P., Suchodolski J.S., Oyama M.A. (2021). Gut Dysbiosis and Its Associations with Gut Microbiota-Derived Metabolites in Dogs with Myxomatous Mitral Valve Disease. mSystems.

[28] Staley C., Weingarden A.R., Khoruts A., Sadowsky M.J. (2017). Interaction of gut microbiota with bile acid metabolism and its influence on disease states. Appl. Microbiol. Biotechnol..

[29] Weisel M.K., Powers J.D., Powers T.E., Baggot J.D. (1977). A pharmacokinetic analysis of tylosin in the normal dog. Am. J. Vet. Res..

[30] Kilpinen S., Spillmann T., Westermarck E. (2014). Efficacy of two low-dose oral tylosin regimens in controlling the relapse of diarrhea in dogs with tylosin-responsive diarrhea: A prospective, single-blinded, two-arm parallel, clinical field trial. Acta Vet. Scand..

[31] Cabral D.J., Penumutchu S., Reinhart E.M., Zhang C., Korry B.J., Wurster J.I., Nilson R., Guang A., Sano W.H., Rowan-Nash A.D. (2019). Microbial Metabolism Modulates Antibiotic Susceptibility within the Murine Gut Microbiome. Cell Metab..

[32] Sommer F., Anderson J.M., Bharti R., Raes J., Rosenstiel P. (2017). The resilience of the intestinal microbiota influences health and disease. Nat. Rev. Microbiol..

[33] Bogatyrev S.R., Rolando J.C., Ismagilov R.F. (2020). Self-reinoculation with fecal flora changes microbiota density and composition leading to an altered bile-acid profile in the mouse small intestine. Microbiome.

[34] Bo T.-B., Zhang X.-Y., Kohl K.D., Wen J., Tian S.-J., Wang D.-H. (2020). Coprophagy prevention alters microbiome, metabolism, neurochemistry, and cognitive behavior in a small mammal. ISME J..

[35] Tal M., Verbrugghe A., Gomez D.E., Chau C., Weese J.S. (2017). The effect of storage at ambient temperature on the feline fecal microbiota. BMC Vet. Res..

[36] Tedjo D.I., Jonkers D.M., Savelkoul P.H., Masclee A.A., van Best N., Pierik M.J., Penders J. (2015). The effect of sampling and storage on the fecal microbiota composition in healthy and diseased subjects. PLoS ONE.

[37] Quévrain E., Maubert M.A., Michon C., Chain F., Marquant R., Tailhades J., Miquel S., Carlier L., Bermúdez-Humarán L.G., Pigneur B. (2016). Identification of an anti-inflammatory protein fromFaecalibacterium prausnitzii, a commensal bacterium deficient in Crohn’s disease. Gut.

